# Knockout of NPFFR2 Prevents LPS-Induced Depressive-Like Responses in Mice

**DOI:** 10.3390/ijms22147611

**Published:** 2021-07-16

**Authors:** Zachary Yu, Ya-Tin Lin, Jin-Chung Chen

**Affiliations:** 1 Department of Medicine, Chang Gung University, Taoyuan 333, Taiwan; uchiha1zachary2runner@gmail.com; 2 Department of Physiology and Pharmacology, Graduate Institute of Biomedical Sciences, Chang Gung University, Taoyuan 333, Taiwan; qpilipalaq@gmail.com; 3 Healthy Aging Research Center, Chang Gung University, Taoyuan 333, Taiwan; 4 Neuroscience Research Center, Chang Gung Memorial Hospital, Taoyuan 333, Taiwan

**Keywords:** neuropeptide FF, NPFFR2, depression, serotonin 1A receptor (5-HT1AR), LPS

## Abstract

The precise neural mechanisms underlying the pathogenesis of depression are largely unknown, though stress-induced brain inflammation and serotonergic plasticity are thought to be centrally involved. Moreover, we previously demonstrated that neuropeptide FF receptor 2 (NPFFR2) overexpression provokes depressive-like behaviors in mice. Here, we assess whether NPFFR2 is involved in priming of depressive-like behaviors and downregulation of serotonergic 1A receptor (5HT1AR) after lipopolysaccharide (LPS) treatment. The forced swimming test (FST) and sucrose preference test (SPT) were used to quantify depressive-like phenotypes in wild-type (WT) and NPFFR2-knockout (KO) mice. A single dose of LPS (i.p. 1 mg/kg) readily caused increases in toll-like receptor 4 and tumor necrosis factor-α along with decreases in 5-HT1AR mRNA in the ventral hippocampus of WT mice. Furthermore, LPS treatment of WT mice increased immobility time in FST and decreased sucrose preference in SPT. In contrast, none of these effects were observed in NPFFR2-KO mice. While WT mice injected with lentiviral 5-HT1AR shRNA in the ventral hippocampus displayed an unaltered response after LPS challenge, LPS-challenged NPFFR2-KO mice displayed a profound decrease in sucrose preference when pretreated with 5-HT1AR shRNA. Taken together, these results suggest that NPFFR2 modulates LPS-induced depressive-like behavioral phenotypes by downregulating 5HT1AR in the ventral hippocampus.

## 1. Introduction

Major depressive disorder (MDD) is a complicated neuropsychiatric disorder affecting large portions of the population worldwide [1], with a higher prevalence in women than in men [2]. Despite extensive research efforts over the past few decades, the pathophysiology of depression is still not clearly understood [3,4]. One of the most frequently targeted neurochemical substrates implicated in the pathophysiology and development of depression is a deficiency in synaptic serotonin availability in the central nervous system (CNS) [5,6,7]. Accordingly, researchers have developed many promising pharmacological agents to target serotonergic metabolic pathways, including selective serotonin reuptake inhibitors (SSRIs) and specific serotonin receptor antagonists, and these drugs are frequently used to treat patients afflicted with MDD [8,9]. Of the 14 serotonin receptor subtypes, a growing body of evidence supports the central involvement of serotonergic 1A receptor (5-HT1AR) in depression [10,11,12,13,14]. Expression patterns for 5-HT1AR protein can be subdivided into two groups according to synaptic location, i.e., presynaptical autoreceptors found mostly in dorsal raphe nuclei and postsynaptical heteroreceptors, which are highly expressed in hippocampus [15,16]. Autoreceptors and heteroreceptors are thought to exert opposing actions on synaptic serotonin availability in response to agonist treatment. As such, activation of the 5-HT1A autoreceptor may inhibit serotonin release to mitigate SSRI-produced anti-depressive effects [10], while activation of the 5-HT1A heteroreceptor may be responsible for the anti-depressive effects of most, if not all, SSRIs [11,17]. Paradoxically, nearly 50% of all depressive patients fall into a category of SSRI non-responders [18,19]. This clinical reality presents a major challenge to the serotonin theory of depression, and as a result, researchers have begun to focus on delineating the non-mutually exclusive actions of monoamines, neurotrophic factors and neurogenesis; empirical observations suggest these factors can modulate severity and remission in depressive disorders [10,20]. Thus, a modern view of MDD pathogenesis is that it probably involves not only a wide spectrum of central serotonergic deficits, ranging from synthesis, catabolism and reuptake to receptor malfunction [21,22], but it also results from complicated interactions between serotonin and other neurotransmitters, neuropeptides and neurotrophins [23,24,25].

One line of study focuses on the plausible role of immune system activation in mediating the onset of depression [26,27]. Increased levels of pro-inflammatory cytokines in serum and cerebrospinal fluid (CSF) have been observed in patients with MDD diagnosis and suicide attempts [28,29]. In healthy human volunteers, intravenous injection of lipopolysaccharide (LPS) is found to induce depressed moods [30]. Likewise, depressive behaviors, such as psychological fatigue, depressed mood, anxiety and anorexia, may be induced by chronic cytokine treatment of patients with hepatitis; interestingly, these symptoms can be alleviated by paroxetine, a frequently prescribed SSRI [31,32]. Moreover, several meta-analyses have revealed an association between elevated pro-inflammatory cytokine titers and depressive symptoms [29,33,34]. Finally, it has been reported that pre-exposure to chronic mild stress may enhance the levels of toll-like receptor 4 (TLR4), an LPS receptor, in brain tissues [35]. Thus, there is mounting evidence that inflammation may be closely associated with depression, and in this context, depressive symptoms can somehow be modulated by serotonergic action.

In a frequently used inflammation-induced depression model, peripheral administration of LPS to rodents induces sickness and depressive-like behaviors [36,37]. Sickness behavior is characterized by malaise, fever, anorexia and fatigue, which typically resolves within 24 h following the LPS treatment [36]. However, after the resolution of sickness behavior, the rodents persistently display depressive-like behaviors, indicated by increased immobility in the forced swimming test (FST) and anhedonia in the sucrose preference test (SPT) [36,38,39,40]. It has been proposed that neuroinflammation-related activation of TLR4/Myd88/NF-κB signaling pathway in the hippocampus might play an important role in partially mediating LPS-induced depression-like behaviors [41,42]. Several reports have supported the notion that inhibiting cytokine release and/or interrupting the TLR4/Myd88/NF-κB signaling pathway may be effective in reducing the magnitude of LPS-induced depression-like behavior, but not LPS-induced sickness [42,43,44]. Taken together, the results of these studies have two major implications. First, the underlying mechanisms of LPS-induced depression-like behavior and sickness behavior are probably different. Second, hippocampal responses to immune activation may be involved in LPS-induced depression-like behavior.

Neuropeptide FF (NPFF) is a pain-modulating peptide that is expressed in the CNS of all mammals [45]. Previously, we reported that stress may evoke expression of NPFF in the mouse hippocampus and that local activation of its type 2 receptor, NPFFR2, may consequently prime the animal for stress-provoked depressive-like behaviors [46]. Furthermore, we have shown that the hippocampal serotonin level is significantly lower in NPFFR2-overexpressing transgenic mice and in mice treated with NPFFR2 agonist, CFMHC, as compared to wild-type (WT) and vehicle-treated littermates (Figure S1). In this study, we further evaluated the effects of NPFFR2 on LPS-induced ventral hippocampal immune activation and depressive-like phenotypes, using WT and NPFFR2-knockout (KO) mice. After identifying a potential role for serotonin receptor 5HT1AR in modulating NPFFR2-mediated immune activation and depressive-like phenotypes, we confirmed this function by pretreating WT and NPFFR2-KO mice with lentivirus encoding 5-HT1AR shRNA or LacZ shRNA. Overall, our results led us to conclude that silencing of NPFFR2 may prevent the development of LPS-induced depressive-like behaviors by regulating ventral hippocampal immune activation responses and/or 5HT1AR expression.

## 2. Results

### 2.1. LPS Induces Depressive-Like Behaviors in WT but Not NPFFR2-KO Mice

Two groups of mice (WT and NPFFR2-KO) were treated with saline or LPS (1 mg/kg; intraperitoneal, i.p.) 24 h prior to evaluation in the SPT or FST. Decreases in sucrose preference and increases in immobility time were evident in LPS-treated WT mice, but no such effects were observed in NPFFR2-KO mice.

**Figure 1:**
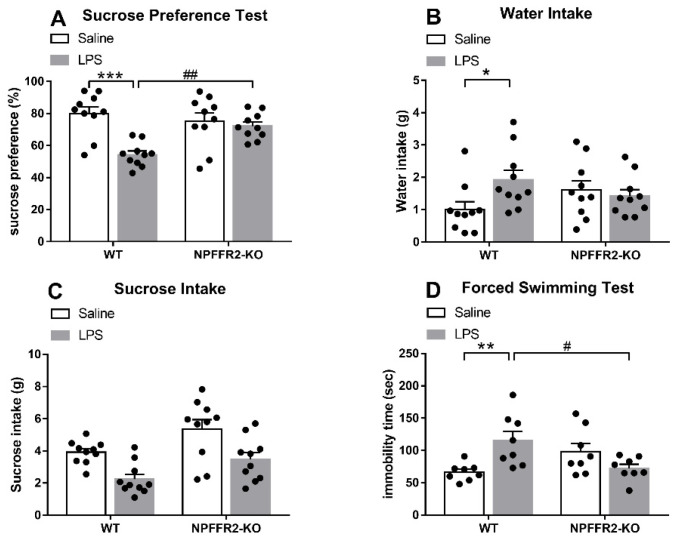
LPS induces depressive-like behaviors in WT but not NPFFR2-KO mice. (**A**) Sucrose preference of saline- and LPS-treated WT and NPFFR2-KO mice. Sucrose preference is calculated according to the following formula: sucrose intake (g)/[sucrose intake (g) + water intake (g)] × 100%. (**B**) Water intake of saline- and LPS-treated WT and NPFFR2-KO mice. (**C**) Sucrose intake of saline- and LPS-treated WT and NPFFR2-KO mice. (**D**) Immobility time of saline- and LPS-treated WT and NPFFR2-KO mice in the forced swimming test. The data are expressed as mean ± S.E.M. and analyzed by two-way ANOVA with Bonferroni post hoc tests. N = 8–10 per group. *, *p* < 0.05; **, *p* < 0.01; ***, *p* < 0.001, comparison between saline and LPS-treated WT mice. #, *p* < 0.05; ##, *p* < 0.01, comparison between LPS-treated WT and NPFFR2-KO mice. Abbreviations: WT, wild type; KO, knockout; LPS, lipopolysaccharide.

As shown in Figure 1A, a two-way ANOVA revealed a significant effect of genotype [F(1,36) = 14.85, *p* = 0.0005] and a significant interaction effect (WT vs. NPFFR2-KO × saline vs. LPS) [F(1,36) = 9.156, *p* = 0.0046] on sucrose preference in the SPT. Bonferroni post hoc tests further showed that LPS-treated WT mice displayed decreased sucrose preference compared to saline-treated controls (*p* < 0.0001). LPS-treated WT mice were found to display significantly lower sucrose preference compared to LPS-treated NPFFR2-KO mice (*p* = 0.0035). Moreover, a two-way ANOVA showed a significant interaction effect (WT vs. NPFFR2-KO × saline vs. LPS) [F(1,36) = 4.682, *p* = 0.0372] on water consumption throughout the SPT (Figure 1B). Bonferroni post hoc tests further revealed that LPS-treated WT mice had higher water intake than their saline-treated counterparts (*p* = 0.0319) (Figure 1B). However, no significant interaction effect of WT vs. NPFFR2-KO × saline vs. LPS was found with regard to sucrose intake (Figure 1C). In the FST, a two-way ANOVA showed that there was a significant interaction (WT vs. NPFFR2-KO × saline vs. LPS) on immobility time [F(1,28) = 13.29, *p* = 0.0011] (Figure 1D). Bonferroni post hoc tests revealed that LPS-treated WT mice had significantly greater immobility times than saline-treated counterparts (*p* = 0.0043). Moreover, LPS-treated NPFFR2-KO mice demonstrated significantly lower immobility times than LPS-treated WT mice (*p* = 0.0122) (Figure 1D).

These results suggest that LPS treatment induces depressive-like behaviors in WT mice, as indicated by sucrose preference decrease in SPT and immobility time increase in FST. However, the depressive-like behaviors are not observed in NPFFR2-KO mice, suggesting that NPFFR2 may be required for LPS-induced depressive-like behaviors.

### 2.2. LPS Induces Ventral Hippocampal Inflammation Responses and 5-HT1AR Changes in WT but Not NPFFR2-KO Mice

To test our hypothesis that NPFFR2 may play a role in mediating LPS-induced 5-HT1AR downregulation and inflammation-related responses in the ventral hippocampus, the mRNA levels of 5-HT1AR, TLR4 and tumor necrosis factor-α (TNF-α) were assessed by real-time PCR. The ventral hippocampal 5-HT1AR mRNA level was decreased in LPS-treated WT mice, while TLR4 and TNF-α mRNA levels were increased. In contrast, the mRNA levels of ventral hippocampal 5-HT1AR, TLR4 and TNF-α in NPFFR2-KO mice treated with LPS remained unaltered when compared to saline control.

**Figure 2:**
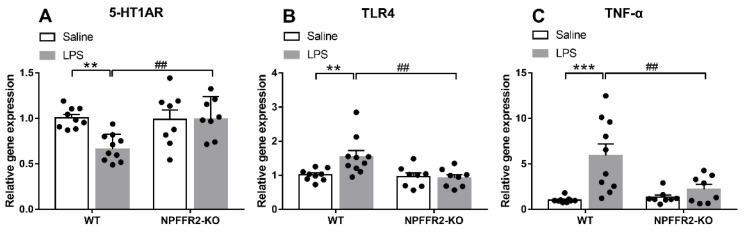
LPS-induced inflammation responses and 5-HT1AR changes in ventral hippocampus of WT and NPFFR2-KO mice. The mRNA levels of (**A**) 5-HT1AR, (**B**) TLR4 and (**C**) TNF-α in saline- and LPS-treated WT and NPFFR2-KO mice are shown. The relative level was calculated by normalizing expression to the saline-treated WT control (fold change). The data are expressed as mean ± S.E.M. and were analyzed by two-way ANOVA with Bonferroni post hoc comparisons. N = 8–10 per group. **, *p* < 0.01; ***, *p* < 0.001; comparisons between saline- and LPS-treated WT mice. ##, *p* < 0.01, comparison between LPS-treated WT and NPFFR2-KO mice. Abbreviations: 5-HT1AR, serotonergic 1A receptor; TLR4, toll-like receptor 4; TNF-α, tumor necrosis factor-α.

A two-way ANOVA revealed that there were significant effects of genotype [F(1,31) = 5.555, *p* = 0.0249], treatment [F(1,31) = 4.973, *p* = 0.0331], and an interaction effect between genotype and treatment [F(1,31) = 6.709, *p* = 0.0145] on 5-HT1AR mRNA level (Figure 2A). Bonferroni post hoc tests further showed that ventral hippocampal 5-HT1AR mRNA was significantly decreased by LPS treatment in WT mice (*p* = 0.0024) (Figure 2A). Moreover, there was a significant difference in the 5-HT1AR mRNA level between LPS-treated WT and NPFFR2-KO mice (*p* = 0.0026) (Figure 2A). Taken together, these findings indicate that ventral hippocampal 5-HT1AR mRNA level was diminished by LPS treatment in WT mice, while such an LPS-dampening effect was not evident in NPFFR2-KO mice. Likewise, a two-way ANOVA revealed significant effects of treatment [F(1,31) = 7.377, *p* = 0.0107] and the interaction between genotype and treatment on TLR4 mRNA levels [F(1,31) = 5.23, *p* = 0.0292] in ventral hippocampus (Figure 2B). Bonferroni post hoc tests showed that ventral hippocampal TLR4 mRNA level was significantly increased by LPS treatment in WT mice (*p* = 0.0067) (Figure 2B). Moreover, there was a significant difference in the TLR4 mRNA level between LPS-treated WT and NPFFR2-KO mice (*p* = 0.0023) (Figure 2B). As shown in Figure 2C, a two-way ANOVA revealed significant effects of genotype [F(1,31) = 13.92, *p* = 0.0008], treatment [F(1,31) = 4.851, *p* = 0.0352] and the interaction of genotype and treatment [F(1,31) = 6.725, *p* = 0.0144] on TNF-α mRNA level in the ventral hippocampus. Bonferroni post hoc tests further showed that the TNF-α mRNA level was significantly increased by LPS treatment in WT mice (*p* = 0.0001) (Figure 2C). Moreover, there was a significant difference in the TNF-α mRNA level between LPS-treated WT and NPFFR2-KO mice (*p* = 0.0034) (Figure 2C).

Together, the results support the notion that LPS treatment of mice causes depressive-like behaviors that coincide with decreased 5-HT1AR mRNA level and enhanced levels of the inflammation-related markers, TLR4 and TNF-α, in the ventral hippocampus. Since NPFFR2-KO mice failed to display these changes, we conclude that NPFFR2 may be at least partially responsible for mediating the LPS-induced inflammatory responses and the decrease of 5-HT1AR mRNA transcripts.

### 2.3. Intra-Ventral Hippocampal 5-HT1AR shRNA Pretreatment Restores LPS-Induced Depressive-Like Behavior in NPFFR2-KO Mice but Has No Effect in WT Mice

**Figure 3:**
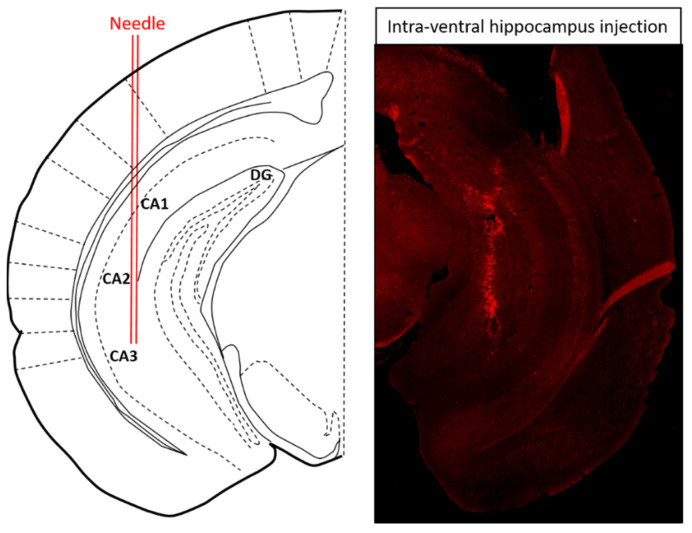
Schematic diagram and representative brain section illustrating the site of intra-ventral hippocampal injection. Seven days after ventral hippocampal injection with adeno-associated virus encoding green fluorescent protein, the mouse was perfused and the brain was fixed by 4% paraformaldehyde and cryosectioned to 25 μm. The injection site for ventral hippocampus was: AP, −3.10 mm; ML, ±3.00 mm and DV, −4.00 mm to bregma [47].

To test whether the reduction in 5-HT1AR level participates in LPS-provoked depressive-like behavior, lentivirus encoding LacZ or 5-HT1AR shRNA was injected into the ventral hippocampus of WT and NPFFR2-KO mice (Figure 3). Approximately 3 weeks after the shRNA administration, all mice were challenged with a single dose of LPS (1 mg/kg; i.p.) and underwent SPT 24 h later. Two weeks after the SPT, the mice were treated with another dose of LPS (1 mg/kg; i.p.) and sacrificed after 24 h to collect the ventral hippocampus. The silencing of 5-HT1AR in the ventral hippocampus by 5-HT1AR shRNA was confirmed in WT and NPFFR2-KO mice by real-time PCR. Interestingly, a decrease in sucrose preference was only observed in 5-HT1AR shRNA- and LPS-treated NPFFR2-KO mice, and not in 5-HT1AR shRNA- and LPS-treated WT mice.

**Figure 4:**
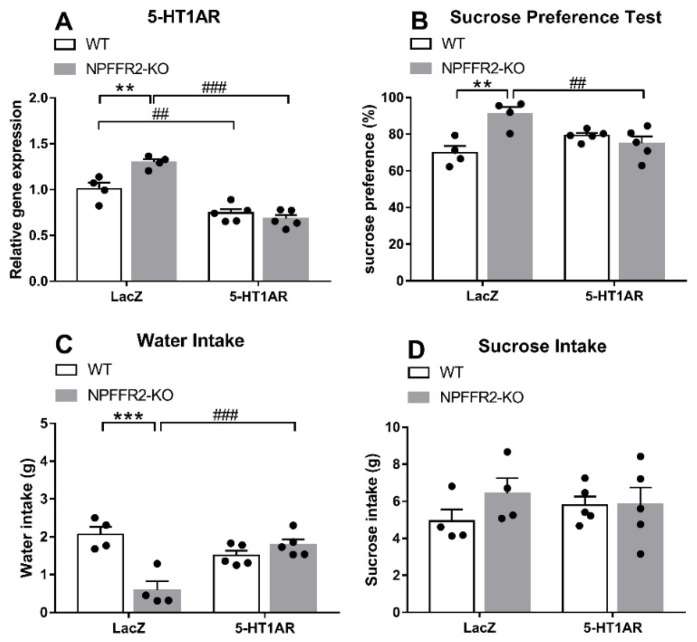
LPS-induced depressive-like behavior in WT and NPFFR2-KO mice pretreated with lentivirus packed-LacZ or 5-HT1AR shRNAs. (**A**) The silencing of 5-HT1AR mRNA by shRNA injection in mouse ventral hippocampus. The relative levels were calculated by normalizing the measurements to the combined LacZ shRNA- and LPS-treated WT mice (fold change). (**B**) Depressive-like behavior as assessed by sucrose preference test. (**C**) Water and (**D**) sucrose solution intake were assessed according to weight consumed (g). The data are expressed as mean ± S.E.M. and were analyzed by two-way ANOVA with Bonferroni post hoc comparison. N = 4–5 per group. **, *p* < 0.01; ***, *p* < 0.001, comparisons between WT and NPFFR2-KO mice treated with combined LacZ shRNA and LPS; ##, *p* < 0.01, ###, *p* < 0.001, comparisons between LacZ and 5-HT1AR shRNA treatment.

As shown in Figure 4A, a two-way ANOVA revealed significant effects of genotype [F(1,14) = 5.78, *p* = 0.0306], shRNA [F(1,14) = 84.53, *p* < 0.0001] and interaction [F(1,14) = 13.56, *p* = 0.0025] on ventral hippocampal 5-HT1AR mRNA level (Figure 4A). Bonferroni post hoc tests further showed that the ventral hippocampal 5-HT1AR mRNA level was significantly decreased by dual 5-HT1AR shRNA and LPS treatments in both WT (*p* = 0.0032) and NPFFR2-KO (*p* < 0.0001) mice (Figure 4A). A significant difference was also detected when comparing the 5-HT1AR levels of LPS-treated WT and NPFFR2-KO mice pretreated with LacZ shRNA (*p* = 0.0022) (Figure 4A). Therefore, our results show that the ventral hippocampal 5-HT1AR mRNA level may be reduced by combined intra-ventral hippocampal 5-HT1AR shRNA and i.p. LPS treatment in both WT and NPFFR2-KO mice (Figure 4A). With regard to the SPT results, a two-way ANOVA revealed significant effects of genotype [F(1,14) = 6.736, *p* = 0.0212] and interaction [F(1,14) = 15.56, *p* = 0.0015] on sucrose preference (Figure 4B). Bonferroni post hoc tests further showed that LacZ shRNA and LPS treatment induced higher sucrose preference in NPFFR2-KO mice as compared with WT mice (*p* = 0.0012) (Figure 4B). Furthermore, there was a significant difference in sucrose preference between LacZ and 5-HT1AR shRNA-pretreated NPFFR2-KO mice (*p* = 0.0067) (Figure 4B). When comparing water intake data shown in Figure 4C, a two-way ANOVA showed significant impact of genotype [F(1,14) = 11.84, *p* = 0.0040] and interaction [F(1,14) = 26.01, *p* = 0.0002] on water intake throughout the SPT. Bonferroni post hoc tests further revealed that combined LacZ shRNA and LPS treatment decreased water intake in NPFFR2-KO mice (*p* = 0.0001) (Figure 4C). Interestingly, there was a significant difference in water intake between 5-HT1AR and LacZ shRNA-pretreated NPFFR2-KO mice (*p* = 0.0005) (Figure 4C). No significant differences were found regarding sucrose intake throughout the SPT (Figure 4D).

These results suggest that knockdown of 5-HT1AR mRNA in the ventral hippocampus allows for the expression of LPS-induced depressive-like behaviors in NPFFR2-KO mice. On the other hand, WT mice with intact NPFFR2 appear to be insensitive to such 5-HT1AR shRNA pretreatment.

## 3. Discussion

In the current study, NPFFR2-KO mice were used to test the hypothesis that NPFFR2 may be involved in mediating LPS-induced mouse depressive-like behaviors. Likewise, female mice were used exclusively for modeling women’s high depression prevalence [48]. We found that LPS injection decreased ventral hippocampal 5-HT1AR mRNA, while increasing neuroinflammation and depressive-like behaviors in WT mice. However, none of these changes were evident in NPFFR2-KO mice. Interestingly, silencing of ventral-hippocampal 5-HT1AR mRNA was found to induce anhedonia in LPS-treated NPFFR2-KO mice. Therefore, the lack of NPFFR2 may protect against LPS-induced depressive-like behaviors through the action of 5-HT1AR downstream signaling.

In recent studies, the role of neuroinflammation in producing depressive symptoms has been rigorously tested in both rodent models and human volunteers [48,49,50,51]. To model neuroinflammation in rodents, peripheral LPS administration is frequently used to elicit a CNS immune response. In particular, peripheral LPS delivery is an effective method to activate hippocampal TLR4 [25,28] among other responses, and the treatment readily stimulates depressive-like behaviors [42,44]. To test whether central neuroinflammation may act through NPFFR2 to induce depression, we used peripheral LPS treatment in this study. In our hands, a single LPS injection produced reliable increases in TLR4 and TNF-α mRNA transcripts in the ventral hippocampus of WT mice 24 h following the injection. Consistently, the WT mice also exhibited prominent depressive-like behaviors, indicated by a decline in sucrose preference and an increase of stress-induced immobility at a similar observation time window.

NPFFR2 is known to be a Gi/o protein-coupled receptor [52], although its intracellular signaling pathways remain mostly undetermined [53]. Notably, NPFFR2 transgenic (Tg) mice are depression-prone and have an over-reactive hypothalamus-pituitary-adrenal axis [46]. Moreover, the depressive-like behaviors of NPFFR2-Tg mice may be counteracted by treatment with a frequently prescribed antidepressant, fluoxetine [46]. In this study, we studied the role of NPFFR2 in mediating LPS-induced depressive-like behaviors by using a NPFFR2-KO mouse model. Recently, we demonstrated that the NPFFR2-KO mouse has a diminished response to a single prolonged stress [54]. Although LPS treatment reliably produced a depressive-like phenotype in WT mice, the treatment did not produce observable depressive-like behaviors (indexed by sucrose preference and immobility time) or ventral hippocampal inflammatory responses (revealed by TLR4 and TNF-α mRNA transcripts) in NPFFR2-KO mice. These findings prompted us to conclude that lack of NPFFR2, presumably in the hippocampus, plays a neuroprotective role from provoked neuroinflammation and depressive-like behavioral phenotypes in LPS-treated animals. However, the pro-inflammatory effect of NPFFR2 in our study stands in stark contrast with a previously identified role of NPFF, its endogenous ligand [55]. NPFF is thought to exhibit anti-inflammatory effects, as it significantly attenuates peritoneal macrophage activity [40]. This inconsistency might be explained by the contexts of inflammation in the two studies. In fact, the action of LPS in the body is complex, as it may stimulate proliferation and differentiation of peripheral macrophages as well as CNS microglia and astrocytes [56].

Many lines of evidence support the idea that downregulated hippocampal 5-HT1A heteroreceptor is one of the most relevant biological predictors for the development of depressive-like behaviors and the efficacy of certain antidepressants [11,17,57,58]. Decreases in hippocampal 5-HT1AR have been correlated with the magnitude of depressive-like behavior [59], and the anti-depressant effect of fluoxetine is abolished in mice lacking 5-HT1AR in mature dentate gyrus granule cells [60]. Consistent with these findings, we found that a single dose of LPS provoked depressive-like behaviors and caused a decrease of 5-HT1AR mRNA levels in the ventral hippocampus of WT mice. Moreover, silencing of ventral hippocampal 5-HT1AR with shRNA induced anhedonia (indexed by sucrose preference decline) in the LPS-treated NPFFR2-KO mice. Intriguingly, silencing of ventral hippocampal 5-HT1AR did not affect anhedonia in WT mice. These puzzling findings could be explained by a so-called floor effect of silencing 5-HT1AR mRNA on sucrose preference in LPS-treated WT mice. This potential explanation is especially compelling, considering the fact that LPS-treated WT mice pretreated with LacZ shRNA had the lowest sucrose preference among the four experimental groups. Another possible explanation could be related to the fact that we used exclusively female mice as experimental animals. Sex differences are clearly observed in inflammation-related depression [61]. Female rats display milder depressive-like phenotypes than male rats after LPS administration [62]. Accordingly, the sex-specific effects of ventral hippocampal 5-HT1AR on LPS-provoked depressive-like phenotypes are unknown, and the topic merits further study. The mechanism underlying crosstalk between NPFFR2 and 5-HT1AR signaling is another question raised by our results, and it too warrants further study. It was of interest to note that following LPS treatment, LacZ-shRNA-treated NPFFR2-KO mice seemed to display less water intake than LacZ-shRNA-treated WT mice (Figure 4C), while such effect was marginal in NPFFR2-KO vs. WT mice treated with LPS (Figure 1B). Nonetheless, LPS-treated WT mice in these two experiments demonstrated comparable water intakes (approximately 2 g), regardless of intra-ventral hippocampal infusion with LacZ-shRNA. Moreover, LPS-treated NPFFR2-KO mice exhibited slightly higher sucrose, while lower water, intake as compared to the LPS-treated WT mice across two experiments. Therefore, intra-ventral hippocampal LacZ-shRNA infusion, at best, minor affect mice’ fluid intake motivation and/or behavior.

In summary, our data suggest that NPFFR2 may act through 5HT1AR to modulate LPS-induced ventral hippocampal neuroinflammation and depressive-like performance in various tests. The lack of NPFFR2 appears to prevent LPS-treated animals from exhibiting LPS-induced ventral hippocampal inflammation and depressive-like behaviors. Furthermore, the unaffected function of ventral hippocampal 5-HT1AR in NPFFR2-deficient mice is likely responsible for protecting animals against LPS-induced depressive-like behaviors.

## 4. Materials and Methods

### 4.1. Animals

All animals were bred in an SPF facility and acclimatized to the animal room, which had a controlled temperature and humidity with a 12 h day-night cycle (light on at 7:00 a.m.). C57BL/6 mice (5 months old, 20–23 g) were housed in 4–5 per cage, with food and water available ad libitum. All the mice used in this study were female. Generation of NPFFR2-KO mice was based on the CRISPR/Cas9 technique and the strain was maintained as homozygotes. Experimental details were described previously [54]. Animal handling and drug treatments were performed in strict accordance with the NIH Guide for the Care and Use of Laboratory Animals. All procedures were approved by the Animal Care Committee of Chang-Gung University (CGU 107-100) in an Association for Assessment and Accreditation of Laboratory Animal Care International (AAALAC)-accredited facility.

### 4.2. LPS-Induced Depressive-Like Behaviors

LPS (L-3129, serotype 0127:B8, SIGMA, St. Louis, MO, USA) solution was freshly prepared on the day of injection by dissolving the compounds in sterile saline. The NPFFR2-KO and WT mice received an i.p. injection (5 μL/g) of LPS (1 mg/kg) or saline. Approximately 24 h after LPS or saline administration, the SPT or FST were conducted using two batches of mice to avoid interference. SPT was conducted without previous water deprivation. Mice were allowed free access to two water bottles, one bottle of 2% sucrose and another bottle of tap water for 18 consecutive hours (19:00–13:00). Mouse sucrose preference was calculated according to the following formula: sucrose intake (g)/[sucrose intake (g) + water intake (g)] × 100%. In the FST, mice were individually placed into a water cylinder (15 cm diameter, 50 cm height) filled to a 30-cm depth with 25 ± 2 °C tap water. Immobility time (excludes the time spent climbing and swimming) during the 6-min testing session was calculated from videotape replay.

### 4.3. LPS-Induced Ventral Hippocampal Inflammation Responses and 5-HT1AR Changes

The ventral hippocampi of NPFFR2-KO and WT mice were collected to analyze the LPS-induced 5-HT1AR expression and inflammation-related responses, including TLR4 and TNF-α. To avoid behavior confounding the inflammatory marker and 5-HT1AR assays, mice used in this experiment did not undergo previous FST or SPT. Twenty-four hours after i.p. injection of LPS (1 mg/kg) or saline, the NPFFR2-KO and WT mice were sacrificed to collect brain tissues. Ventral hippocampus was obtained to assay mRNA levels. Total RNA was extracted using TRIzol^®^ reagent (Invitrogen, Carlsbad, CA, USA) according to the manufacturer’s protocol. The mRNA was then reverse transcribed into cDNA via reverse transcription-PCR (GScrtip, GeneDirex, Lais Vegas, NV, USA). The cDNA levels of corresponding targets, including 5-HT1AR, TLR4, TNF-α and the housekeeping gene, Rpl35a, were quantified using a real-time PCR detection system (CFX 96, Bio-Rad, Hercules, CA, USA) and SYBR (Bio-Rad). The results were processed with CFX Manager Software (Bio-Rad). Threshold cycle, which inversely correlates with mRNA level, was measured by the software. The Rpl35a gene served as an internal control. The results were calculated with delta-delta Ct method and normalized to the corresponding control. The primer sequences used in this study are listed in Table 1.

### 4.4. Lenti-5-HT1AR shRNA

The five lentivirus-packaged mouse 5-HT1AR-shRNA constructs were purchased from the National RNAi Core Facility at Academy Sinica of Taiwan (http://rnai.genmed.sinica.edu.tw, accessed on 19 January 2021). LacZ-shRNA served as a mock control, as the shRNA target sequence is not present in mice. Mice were anaesthetized with i.p. injection of a mixture of Zoletil (30 mg/kg) and Rompun (12 mg/kg) and then fixed onto a mouse stereotaxic instrument (David Korf Instrument, Tujunga, CA, USA). A mixture of five types of 5-HT1AR shRNA or a single LacZ-shRNA (1–3 × 10^6^ RIU/mL) were injected bilaterally into the ventral hippocampus (AP, −3.10 mm; ML, ±3.00 mm and DV, −4.00 mm to bregma), using a microsyringe pump (1 μL/min for 30 s). In order to prevent leakage, the needle was left in position for 5 min after the injection. Mice were i.p. injected with Ampicillin (4 mg/kg) and Meloxicam (5 mg/kg) for 3 days as postoperative care. Approximately 3 weeks after the shRNA administration, all mice were treated with a single dose of LPS (1 mg/kg, i.p.) and underwent SPT 24 h later. Two weeks after the SPT, the mice were challenged with another dose of LPS (1 mg/kg; i.p.) and their ventral hippocampal tissues were collected 24 h after the injection. The corresponding clone IDs and interference sequences for the shRNA constructs are listed in Table 2.

### 4.5. Statistical Analysis

Data are expressed as mean ± standard error of mean (S.E.M.). Statistical analyses were performed using GraphPad Prism 7 software. Differences were tested with two-way ANOVA followed by Bonferroni post hoc comparison or unpaired Student’s *t*-test as appropriate. Statistical significance was set as *p* < 0.05.

## Figures and Tables

**Table 1:** Primer sequences.

Target Genes	Forward Primer (5′ to 3′)	Reverse Primer (5′ to 3′)
5-HT1AR	CATGCTGGTCCTCTATGGGC	GGCTGACCATTCAGGCTCTT
TLR4	ATGGCATGGCTTACACCACC	GAGGCCAATTTGTCTCCACA
TNF-α	CCGATGGGTTGTACCTTGTC	GTGGGTGAGGAGCAGTAGT
Rpl35a	GCTGTGGTCCAAGGCCATTTT	CCGATTACTTTTCCCCAGATGAC

**Table 2:** Interference sequences of shRNAs.

Target Gene	Clone ID	Interference Sequences
5-HT1AR	TRCN0000221271	CCCTTCCTGTTCACTCAATAT
5-HT1AR	TRCN0000221272	CGACCCTATAGACTACGTGAA
5-HT1AR	TRCN0000221273	CCTGAGTTGTTGGGTGCCATA
5-HT1AR	TRCN0000221274	GTACACCATCTACTCCACTTT
5-HT1AR	TRCN0000221275	CCCTGCTCAACCCAGTTATTT
LacZ	TRCN0000072232	CGTCGTATTACAACGTCGTGA

## Data Availability

The data presented in this study are available on request from the corresponding author.

## Supplementary Materials

The following are available online at https://www.mdpi.com/article/10.3390/ijms22147611/s1.
