# Supplementary material for: Knockout of NPFFR2 Prevents LPS-Induced Depressive-Like Responses in Mice
Source: Int J Mol Sci. 2021 Jul 16;22(14):7611. doi: 10.3390/ijms22147611 (PMC8306864; doi:10.3390/ijms22147611)
Supplement: Supplementary file 1 [file ijms-22-07611-s001.zip › ijms-1271002-supplementary.pdf]

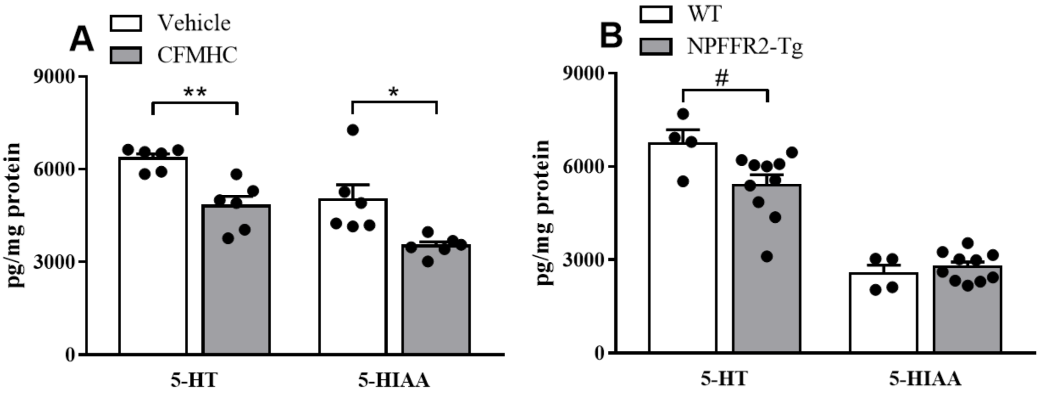

Figure S1. Effects of NPFFR2 activation on serotonin (5-HT) and its metabolite 5-HIAA in the hippocampus of WT and NPFFR2 transgenic (Tg) mice. (A) Effect of NPFFR2 agonist CFMHC treated at 20 mg/kg continuously for 21 days in WT mice. (B) Comparison between NPFFR2 Tg and WT mice. The data are expressed as mean ± S.E.M. and were analyzed by unpaired Student's *t* test. N = 4-10 per group. *, $p < 0.05$; **, $p < 0.01$, comparison between vehicle- and CFMHC-treated WT mice. #, $p < 0.05$, comparison between WT and NPFFR2-Tg mice.
